# Allosteric PROTACs: Expanding the Horizon of Targeted Protein Degradation

**DOI:** 10.1021/jacs.5c14840

**Published:** 2026-01-13

**Authors:** Aileen Frost, Suzanne O’Connor, Alessio Ciulli

**Affiliations:** Centre for Targeted Protein Degradation, School of Life Sciences, 3042 University of Dundee, Dundee DD1 5JJ, United Kingdom

## Abstract

Proteolysis-targeting chimeras (PROTACs) have transformed the concept of chemical intervention in biological systems by co-opting the ubiquitin–proteasome system to selectively degrade proteins. A key promise of this modality is that proximity alone—not inhibition—is required, allowing binding anywhere on the protein surface to trigger degradation. Yet despite this conceptual freedom, most PROTACs to date have been built from orthosteric inhibitors. The use of allosteric or functionally silent ligands remains a largely untapped opportunity. In this Perspective, we spotlight pioneering efforts in allosteric PROTAC design and explore how such strategies could unlock improved outcomes for target selectivity, efficacy, and resistance management while also modulating physicochemical properties to enhance in vivo performance. We further discuss the practical and conceptual challenges and the advances needed to make allosteric targeting a mainstream strategy in the design of protein degraders and other proximity-inducing molecules.

## Graphical abstract

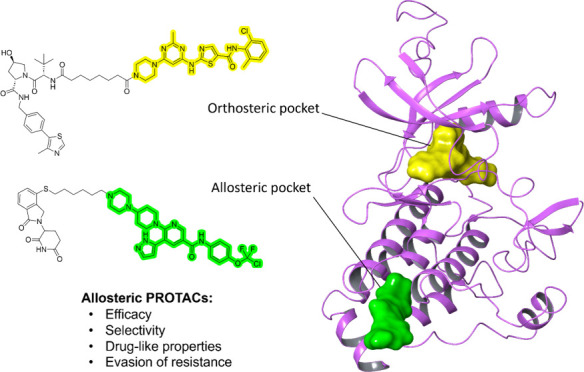

## Introduction

Proteolysis-targeting chimeras (PROTACs) are bifunctional molecules composed of a ligand for a target protein of interest linked to an E3 ligase recruiter.^1^ By forming a ternary complex between the target protein and the ligase, PROTACs induce ubiquitination and subsequent proteasomal degradation. Over the past decade, targeted protein degradation has emerged as a powerful strategy that offers distinct advantages over conventional inhibition.^2,3^ Unlike inhibitors, which typically require high occupancy at functional sites over the duration of the pharmacological action, PROTACs eliminate the entire protein from the cell, thereby disrupting both catalytic and noncatalytic functions and necessitating full resynthesis to restore activity.

The catalytic, substoichiometric mode of action of PROTACs underpins their unique pharmacology. Degradation can be achieved at subsaturating occupancy, and virtually any binding site on a protein can, in principle, serve as a recruiting anchor. This opens opportunities to target proteins considered difficult to drug, and indeed PROTACs have shown the ability to degrade drug-resistant proteins^4^ and discriminate between closely related paralogues even when built from nonselective ligands.^5,6^ At the same time, their design, synthesis and evaluation require significant multidisciplinary expertise in order to develop suitably qualified chemical tools and lead compounds for clinical investigation. Certain target classes – for example, rapidly turned-over i.e. short-lived proteins – can present unique challenges.^7^

Despite the theoretical freedom to bind anywhere on the protein surface, the overwhelming majority of PROTACs reported to date have been derived from orthosteric inhibitors. This reliance on functional active-site binders was understandable in the early days of the field, when proof-of-concept degraders were needed to establish the approach. However, the field has now matured to a point where the potential of allosteric and functionally silent ligands should be more fully explored.

In this Perspective, we focus on “allosteric PROTACs,” defined here as degraders whose target-binding moiety engages the target protein outside of its orthosteric site, regardless of whether that ligand has intrinsic functional activity. We also include PROTACs built from nonfunctional ligands, regardless of whether binding to allosteric or orthosteric pockets, as we believe these are an important resource for the development of new degraders. We highlight how such ligands have been discovered, how they have been repurposed for degrader design, and the specific advantages they can offer. In particular, we explore how allosteric PROTACs may help overcome resistance mechanisms, achieve isoform and paralogue selectivity, enhance pharmacological efficacy, and improve physicochemical properties for in vivo applications.

### Allosteric PROTACs Can Help Evade Resistance Mechanisms

Resistance to inhibitors is unfortunately common, particularly with kinase inhibition, and leads to loss of efficacy in patients who rapidly relapse following initial response. Resistance can occur via multiple mechanisms including upregulation of the target protein via feedback loops, pathway rewiring or mutations in the binding site that lower the binding affinity of the drug by steric clashing or removing key protein–ligand interactions.^8^ Allosteric PROTACs targeting EGFR, MEK and BCR-ABL have been developed from allosteric kinase inhibitors and have shown to be effective against these resistance mechanisms.

### EGFR

Gefitinib, erlotinib, afatinib, dacomitinib and osimertinib are orthosteric epidermal growth factor receptor (EGFR) inhibitors that have been clinically approved for the treatment of non-small cell lung cancer, but resistance rapidly arises from mutations within the ATP binding site, compromising their efficacy. While the third generation irreversible inhibitor osimertinib is active against the T790M mutant,^9^ acquired resistance occurs by mutation of the key cysteine residue it binds to, C797.^10^ Allosteric EGFR ligands were developed to potentially overcome drug resistant ATP binding site mutations that arise from orthosteric inhibitors.^11^ A library of 2.5 million compounds was screened using a biochemical assay with purified EGFR (L858R/T790M) and 1 mM ATP, counter screening active compounds with 1 mM ATP and against wild type EGFR to identify compounds that were both non-ATP competitive and selective over the wild type EGFR. This screen identified a hit compound EAI001 which was optimized to EAI045, a 3 nM L858R/T790M mutant inhibitor with approximately 1000-fold selectivity over wild-type EGFR. However, this compound was found to be inactive as a single agent and required combination with the monoclonal antibody cetuximab as a dimer disrupting agent to be effective in cell proliferation assays.

Using the X-ray crystal structure of the allosteric ligand EAI001, the compound was extended further into the solvent exposed region to make compound JBJ-07–149, providing a suitable exit vector for PROTAC synthesis (Figure 1). Attaching a variety of PEG linkers to the piperazine of JBJ-07–149 and the CRBN binder pomalidomide gave a series of allosteric EGFR degraders, of which DDC-01–163 was the most potent analogue in antiproliferation assays.^12^ DDC-01–163 inhibits the mutant EGFR L858R/T790M Ba/F3 cell line selectively over the wild-type EGFR Ba/F3 cell line and was found to be effective against osimertinib resistant cells with L858R/T790M/C797S and L858R/T790M/L718Q EGFR mutations. Additionally, treating L858R/T790M Ba/F3 cells with the allosteric PROTAC DDC-01–163 in combination with the orthosteric EGFR inhibitor osimertinib enhanced the observed antiproliferative effect of the PROTAC alone. It has since been shown crystallographically that the orthosteric inhibitor osimertinib can bind simultaneously with the allosteric inhibitor EAI045 (Figure 1) supporting the use of a combination strategy to tackle EGFR resistance mechanisms.^13^

**Figure 1:**
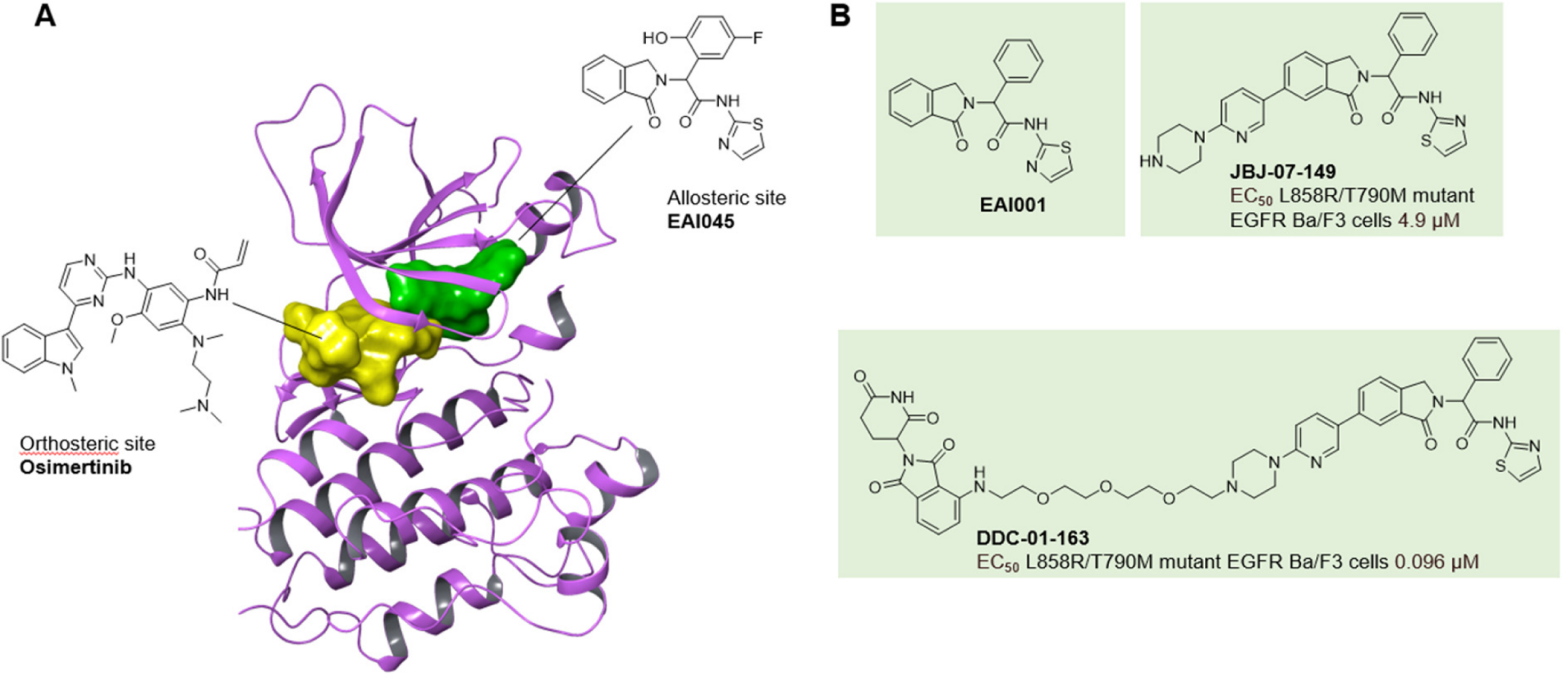
Allosteric PROTACs can help to evade resistance mechanisms. (A) X-ray crystal structure (PDB 6Z4B) of the allosteric ligand EAI045 (green) and the orthosteric ligand osimertinib (yellow) bound simultaneously to EGFR T790M. (B) Allosteric EGFR inhibitors EAI001 and JBJ-07–149, and the related allosteric EGFR degrader DDC-01–163, with comparison of inhibition of cell proliferation in Ba/F3 cell lines that were stably transfected with the EGFR L858R/T790M mutant.

### MEK

The MAPK pathway is critical in regulating cell cycle control, including proliferation, differentiation and survival. Aberrant pathway signaling is often implicated in cancers, with mutations contributing to unregulated cellular division. Therefore, members of this signaling cascade have been prime targets for inhibition in attempts to treat MAPK driven cancers.^14–16^

Many allosteric MEK inhibitors have been developed, building on the discovery of PD 098059, the first inhibitor reported to act on the MAPK pathway.^17^ PD 098059 was identified via a screen utilizing an *in vitro* kinase assay measuring phosphorylation of MAPK substrates. Follow-up experiments indicated that MEK inhibition by PD 098059 was acting via an allosteric mechanism, as the phosphorylated forms of MEK were not inhibited and inhibition was found to be noncompetitive with ATP.^18^

Allosteric MEK inhibitors have exhibited cardiovascular^19^ and ocular^20^ toxicity in clinical use, which is a result of their mechanism of action. Upon MEK inhibition, a negative feedback loop driven by p-ERK results in an increase in phosphorylated MEK. In this instance, targeted protein degradation may prove advantageous to overcome this acquired resistance, as removal of MEK via degradation could work to counteract the feedback effect. As many allosteric MEK ligands are known, several examples of allosteric PROTACs targeting MEK have been reported in the literature.^21,22^

Vollmer et al.^23^ have utilized compound **1**, a ligand based on Refametinib to design and synthesize allosteric MEK PROTACs. Refametinib binds to an allosteric site on MEK, holding the protein in the inactive conformation.^24^ A cocrystal structure of Refametinib in MEK informed the choice of exit vector for linker attachment, and both VHL and CRBN-recruiting PROTACs were synthesized (Figure 2). Ultimately, the VHL compounds were found to be better degraders of MEK1 and MEK2, with compound **1** shown as representative. At low treatment concentrations of either PROTAC or inhibitor an increase in MEK protein levels was observed, thought to be due to the negative feedback mechanism which regulates MEK protein levels. Although phospho-ERK inhibition is less potent for PROTACs vs the parent inhibitor, PROTACs all showed stronger antiproliferation activity in A375 cells. The authors postulate that this could be due to a longer lasting effect of degradation vs inhibition, or an added benefit that may come from abrogating scaffolding functions or adaptor proteins of MEK/ERK signaling.

**Figure 2:**
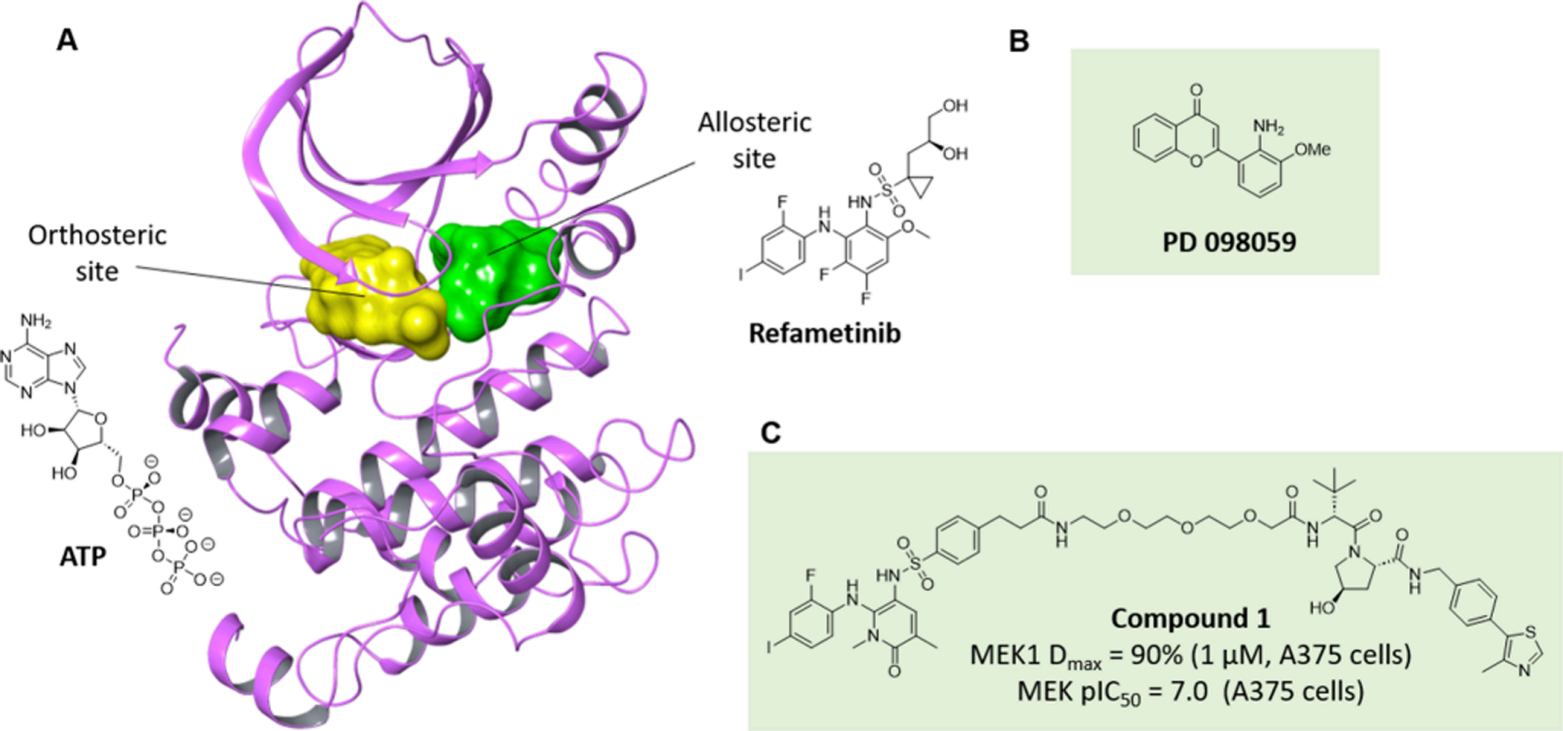
Allosteric PROTACs for MEK. (A) Co-crystal structure of the allosteric MEK inhibitor Refametinib (green) and Mg-ATP (yellow) in MEK1 (PDB 3E8N). The allosteric site is adjacent to the Mg-ATP binding site. Although ATP can still bind in this noncompetitive inhibition mode, binding to ERK is precluded. (B) 2D structure of allosteric ligand PD098059. (C) Allosteric PROTAC compound **1**.

### BCR-ABL

The BCR-ABL (Breakpoint Cluster Region- Abelson Tyrosine Kinase 1) fusion protein is implicated as a key driver of chronic myeloid leukemia (CML).^25^ Initially, inhibition of the ATP-binding site provided a successful treatment option for patients, but resistance mutations have ultimately limited the efficacy of this approach.^26^ In particular, the T315I gatekeeper mutation is resistant to all known orthosteric inhibitors, except ponatinib, which is known to cause adverse vascular events.^27,28^ FDA-approved allosteric inhibitor asciminib binds at the myristoyl binding pocket and is active against most BCR-ABL kinase domain mutations, including T315I.^29,30^ However, resistance to asciminib has also been observed via myristate-site mutations and this allosteric inhibitor shows limited efficacy against highly resistant compound mutations, which contain ≥ 2 mutations in the same Bcr-Abl allele.^32,33^

In an effort to ascertain whether a degradation phenotype can address the acquired resistance to BCR-ABL inhibitors, a number of research groups have pursued BCR-ABL degraders using either orthosteric ligands or allosteric ligands based on GNF-5 or asciminib (Figure 3).^34–38^ Development of the GNF-5 inhibitor began with a differential cytotoxicity screen to identify compounds which could selectively inhibit proliferation of BCR-ABL-dependent cell lines.^39^ Hits were tested for their ability to inhibit recombinant ABL kinase domain activity and cellular BCR-ABL autophosphorylation. Binding of hit compound GNF-2 could not be outcompeted with either ATP or the orthosteric inhibitor imatinib but could with a myristoylated peptide. Confirmation that the myristate binding pocket is the binding site of GNF-2 was achieved by X-ray crystallography. Further medicinal chemistry optimization led to the development of GNF-5. Discovery of the allosteric inhibitor asciminib built on these efforts, employing an NMR fragment screen against ABL1 with the ATP binding site blocked using imatinib. Subsequent optimization efforts were aided by X-ray crystallography and molecular modeling.^31^

**Figure 3:**
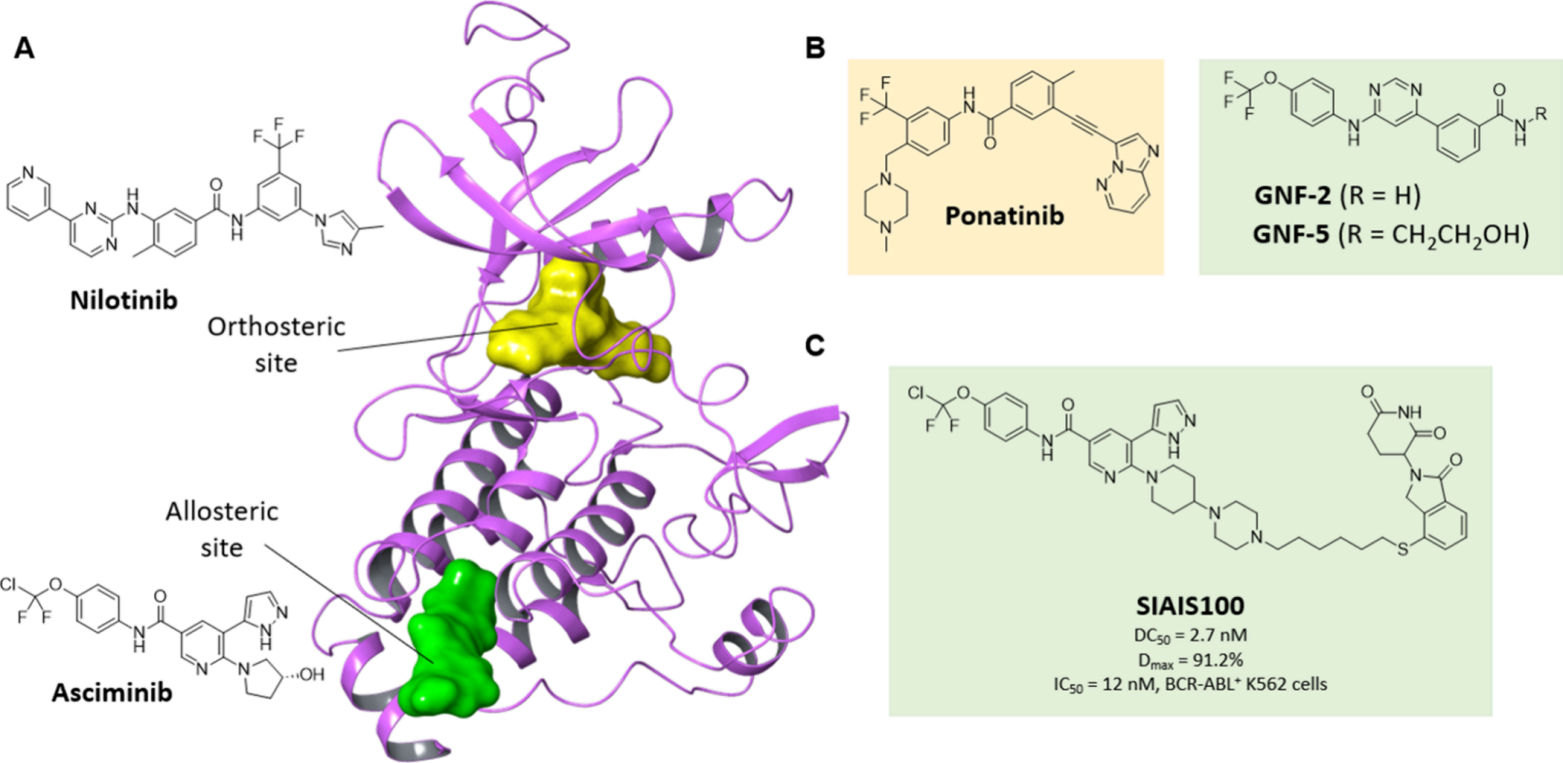
Allosteric BCR-ABL inhibitors and PROTACs. (A) Co-crystal structure of nilotinib and asciminib in ABL kinase (PDB 5MO4). Nilotinib (yellow) binds in the catalytic site, whereas asciminib (green) binds allosterically in the myristoyl binding site (B) Chemical structures of orthosteric inhibitor ponatinib and allosteric inhibitors GNF-2 and GNF-5. (C) Allosteric degrader SIAIS100.

Liu et al. have developed the potent BCR-ABL PROTAC SIAIS100 by utilizing acsiminib as a target protein binder, evaluating its efficacy across ten clinically relevant resistance mutations.^39^ Of these, SIAIS100 was shown to degrade acsiminib-responsive mutants T315I and E255V, but additionally acsiminib-resistant mutations K294E, P223S, T315M, F359V and G671R. Furthermore, degradation of compound mutations G250E/T315I, Y253H/T315I, and Y253H/E255V was achieved, demonstrating a clear benefit over asciminib which is either ineffective or has reduced activity against these challenging mutants. This study provides a great example for where allosteric PROTACs could lead to improved treatment options for patients. However, it should be noted that upon proteomic analysis it was found that SIAIS100 degrades CRBN neo-substrates IZKF1, IZKF3 and ZFP91 in addition to BCR-ABL. Development of a BCR-ABL selective degrader would be important to avoid possible off-target toxicities.

In a complementary study, Burslem and co-workers have reported allosteric BCR-ABL PROTACs which are VHL-recruiting and utilize either GNF-5 or acsiminib. The GNF-5-based PROTAC showed enhanced antiproliferative effects upon cotreatment with an orthosteric inhibitor, which the authors suggested could allow for reduced dosing and therefore modulate side effects. Furthermore, this presents an alternative strategy to addressing acquired resistance. Building upon this work, the same authors published a scaffold-hopping approach in which an acsiminib-based binder was conjugated to the same VHL ligand and linker used in the first study. This resulted in a PROTAC with 10-fold improved degradation potency, and 100-fold more potent antiproliferative effects in K562 cell line. It would be interesting to see further evaluation of this PROTAC against a comprehensive range of BCR-ABL resistance mutations, and a proteomic study to validate its selectivity. This would allow comparison with the acsiminib-based CRBN recruiting PROTAC characterized by Liu et al., and provide insight into the relative merits of CRBN or VHL recruiting allosteric PROTACs in overcoming resistance to BCR-ABL orthosteric inhibitors.

These examples highlight the power allosteric PROTACs can have for overcoming resistance mechanisms including mutations in the ATP binding site or negative feedback loops that can significantly impact the effectiveness of drugs. Improved efficacy over allosteric inhibitors can be achieved, by virtue of the degradation phenotype.

### Allosteric PROTACs Can Afford Greater Target Selectivity

Selectivity for a target of interest is a key consideration when undertaking compound design. Unwanted engagement and activity against any targets with similar binding sites could complicate drug pharmacology and lead to side effects and/or toxicities. For example, designing kinase-targeting drugs to be selective for specific individual protein kinases can be particularly challenging, as in many cases ATP binding sites are highly homologous and structurally similar. Non-ATP binding sites on closely related protein kinases are more likely to bear structural differences, that can be exploited pharmacologically, presenting an opportunity for allosteric inhibitors or PROTACs to address these targets more selectively.

### TYK2

TYK2 is a member of the JAK family of tyrosine kinases that has been investigated as an attractive therapeutic target for a range of autoimmune diseases and cancers.^40^ The catalytic site of TYK2 shares high sequence and structural homology with JAK2, making the development of selective orthosteric inhibitors challenging. As pan JAK inhibitors have undesirable side effects, and JAK2 inhibition has been linked to hematopoietic defects such as anemia, selective TYK2 inhibition would be desirable.^41^ Across the JAK family, binding site residues within the pseudokinase domains are less conserved compared to the catalytic site, allowing greater opportunities for selective binding or inhibition.

An allosteric binding site of TYK2 was discovered via a phenotypic high-throughput screen.^42^ Co-crystallization of an early hit compound showed binding to the pseudokinase domain, which is thought to induce an inactive conformation of the kinase domain, thereby precluding binding of ATP. A cocrystal structure of TYK2 with compound **2**, which binds to both the kinase and pseudokinase domains, shows the relative positions of both orthosteric and allosteric sites (Figure 4). Optimization of this hit ultimately led to the development of the allosteric inhibitor Deucravacitinib, a first in class oral allosteric TYK2 inhibitor that was FDA approved in September 2022 for moderate to severe psoriasis.

**Figure 4:**
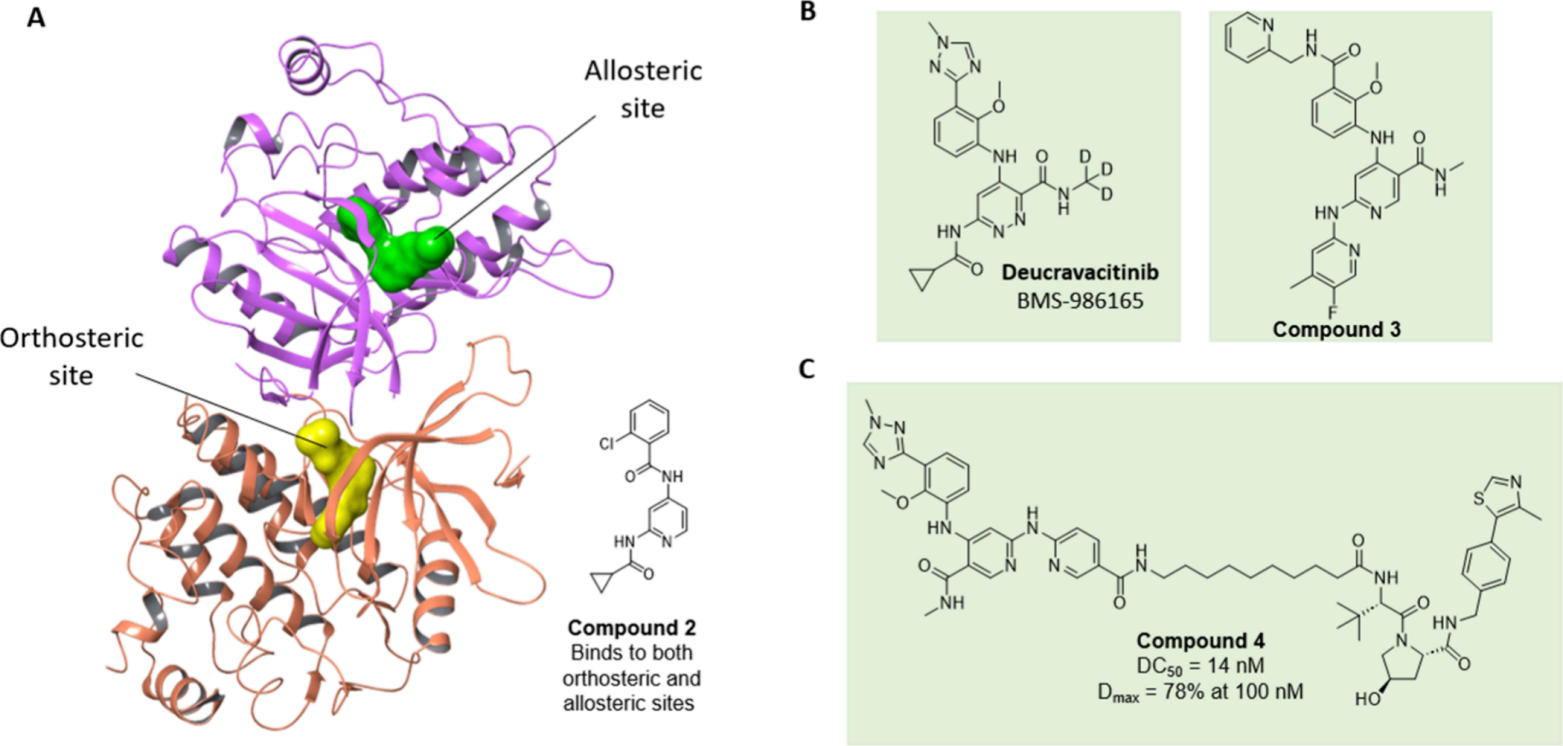
TYK2 inhibitors and PROTACs. (A) Co-crystal of TYK2 JH2 kinase (red) and pseudokinase (purple) domains with compound **2** which binds to both orthosteric (yellow) and allosteric (green) sites. [PDB 4OLI];^44^ (B) Chemical structures of allosteric TYK inhibitor Deucravactinib and allosteric ligand compound **3**, which has been used as a basis for PROTAC design. (C) Chemical structure of compound **4**, with degradation data.

The Deucravacitinib derivative compound **3** was explored as a starting point for PROTAC development.^43^ Analysis of the X-ray crystal structure of compound **3** within the JH2 domain of TYK2 was undertaken to aid PROTAC design. A solvent exposed region was identified from which a selection of alkyl linkers was employed to link a VHL ligand to the allosteric TYK2 ligand. Of the compounds detailed in this study, compound **4** showed optimal TYK degradation potency, while maintaining good selectivity over JAK1 making it a suitable tool compound to study the impact of TYK2 degradation on the biology of the IL3-reporter pathway. Allosteric degradation makes it possible to target the kinase independent scaffolding function of TYK2, while taking advantage of the selectivity afforded over other JAK protein family members.

It has been demonstrated that bifunctional degraders have the potential to overcome selectivity issues between proteins with high sequence homology by virtue of neo-interactions induced within the ternary complex, even when a nonselective orthosteric ligand is utilized. The improved selectivity of allosteric over orthosteric inhibitors is well documented in the kinase field, where ATP-competitive binding sites are highly conserved. Developing allosteric PROTACs from allosteric kinase ligands has the potential to capitalize on this selectivity and to abrogate noncatalytic functions of the target proteins, as in the TYK2 example discussed above. Invoking the use of allosteric ligands for PROTAC development provides further opportunity for differentiation between targets and, therefore, to enhance compound selectivity.

### Allosteric PROTACs Can Demonstrate Improved Efficacy

Allosteric site PROTACs have shown improved efficacy relative to their parent inhibitors, either by virtue of a protein knockout phenotype, for example SHP2, or by exploiting distinct binding modes to expand efficacy across further cell lines/indications as shown for AKT.

### AKT

Inhibition of the serine/threonine kinase AKT (protein kinase B) has been extensively pursued as a potential cancer treatment strategy. Inhibition of AKT with ATP-competitive small molecules has shown toxicity and limited efficacy as single agents in clinical trials, although Capivasertib has been approved in combination with Fulvestrant in specific breast cancer settings. Although allosteric AKT inhibitors have also been developed, they have not yet shown sufficient efficacy in a clinical setting.^45^ In cases like this a PROTAC strategy can be helpful to increase efficacy due to the catalytic mode of action and the option to use ligands that bind outside of the ATP binding site, thus negating the need to compete with ATP. While potent AKT PROTACs derived from a number of different ATP-competitive inhibitors have been reported,^46–50^ Yu et al. have pursued an allosteric PROTAC, which in comparison to their previous orthosteric PROTAC (MS21)^51^ has improved efficacy in KRAS and BRAF mutant cell lines.^52^

Orthosteric and allosteric AKT ligands adopt distinct binding modes, with allosteric ligands inducing a significant shift in protein conformation caused by molecule binding in a pocket which exists between the kinase domain and the PH domain. This results in an autoinhibitory conformation, in which an interaction between the PH domain and the C-lobe displaces the activation loop within the kinase domain outward, burying the phosphoinositide binding region. The ATP binding region is occupied with hydrophobic residues, which preclude the binding of both ATP and ATP-competitive inhibitors (Figure 5).^53–55^

**Figure 5:**
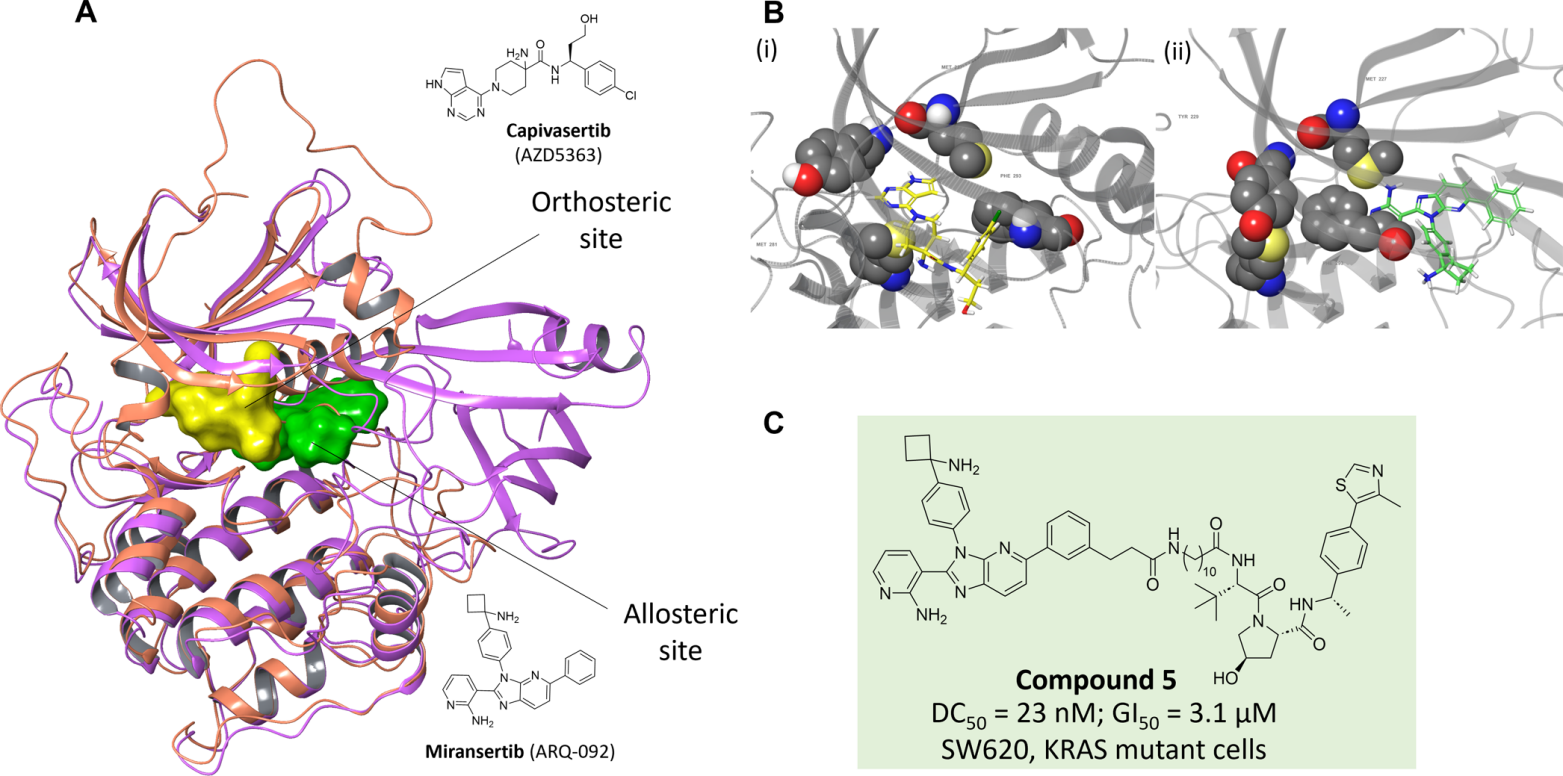
Allosteric AKT inhibitors and PROTACs. (A) Overlay of cocrystal structures (i) orthosteric inhibitor AZD5363 (yellow) in AKT1 (orange) [4GV1] and (ii) allosteric inhibitor ARQ-092 (green) in AKT1 (purple) [5KCV]. Allosteric binding mode promotes significant structural shift. (B) Side-by-side comparison of cocrystal structures of (i) orthosteric inhibitor AZD5363 (yellow) and (ii) allosteric inhibitor ARQ-092 (green) Arrangement of hydrophobic residues in (i) allows for binding of ATP and ATP-competitive inhibitors. Allosteric binding in (ii) promotes a conformational shift which precludes binding of ATP and ATP-competitive inhibitors. (C) Allosteric AKT PROTAC compound **5**.

Ashwell et al. exploited the inactivity of orthosteric AKT inhibitors toward recombinant unphosphorylated AKT to bias their screening efforts toward the discovery of allosteric inhibitors, which would preferentially bind to the autoinhibited conformation.^56^ Compounds were tested against unphosphorylated full length AKT using a biophysical screening cascade involving indirect affinity mass spectrometry (IA-MS), followed by thermal shift assay (TSA) and surface plasmon resonance (SPR) to confirm hits. Controls were undertaken to show that neither an ATP mimetic (AMP-PNP) nor an ATP-competitive inhibitor (A674563) were capable of binding to this construct of AKT. Further optimization of hits from this screen led to the discovery of allosteric AKT inhibitor Miransertib.

Using the cocrystal structure of Miransertib (ARQ-092) a series of allosteric AKT PROTACs was designed by identifying a suitable point for exit vector conjugation. Both VHL and CRBN recruiting PROTACs were synthesized with ligands VHL-1 and pomalidomide respectively, attached via alkyl or PEG linkers of various lengths. VHL PROTACs gave superior degradation and SAR with respect to linker length showed that alkyl linkers with a 7–10 carbon chain were most effective. Tuning the affinity of the VHL ligand by adding a benzylic methyl group in compound **5** improved degradation potency.^57^

Compound **5** performed well in KRAS/BRAF mutant cell lines which exhibit low phosphorylated AKT levels, showing enhanced activity over previously reported orthosteric PROTAC MS21. Further studies in SW620 cells confirmed PROTAC mechanism of action and showed strong antiproliferative effects, similar to the parent inhibitor Miransertib ARQ-092. Exploitation of the distinct binding mode of allosteric AKT ligands here presents a way to increase degrader efficacy across a wider range of cell lines, thus improving the scope for this strategy for future studies or to inspire treatment options.

### SHP2

SHP2 (Src homology 2 domain-containing phosphatase 2) is implicated in multiple cancer signaling pathways, including the RAS-RAF-MEK-ERK pathway.^58^ Inhibition of SHP2 in this context decreases phosphoERK levels, leading to apoptosis of cancer cells. Furthermore, CRISPR-Cas-9 inactivation of SHP2 leads to impaired growth of KRAS mutant tumors^59^ and knockout of the gene which encodes SHP2, *PTPN-11*, leads to reduced proliferation in PDAC cells.^60^ Efforts to target SHP2 via orthosteric inhibition have failed to show therapeutic efficacy due to insufficient cellular activity or selectivity. Moreover, the highly solvated and polar nature of SHP2’s catalytic site has meant that orthosteric inhibitors have unfavorable physicochemical properties, which limits their use.^61^

The development of the allosteric SHP2 inhibitor SHP099 by Novartis represented a significant breakthrough, exhibiting SHP2 selectivity and efficacy in xenograft models. Discovery of SHP099 was achieved via a biased screening approach, taking advantage of a natural regulatory mechanism whereby SHP2 is locked in an autoinhibited conformation^62,63^ Identification of allosteric inhibitor hits was achieved by discarding compounds which were found to inhibit phosphatase activity while also binding to the PTP domain of SHP2. Further optimization of hits led to clinical candidate SHP099. Building on this work, several SHP2 allosteric inhibitors have entered clinical trials, however, none have been approved to date.

Protein degradation is a better pharmacological mimic of protein knockdown e.g. via genetics or RNAi, thus it is expected that SHP2 target degradation should be more efficacious than target inhibition. Wang et al. have capitalized on this to develop allosteric SHP2 PROTACs (Figure 6).^64^ Using designs inspired by SHP099 and other known SHP2 binders, a sulfur atom was introduced between the two aromatic rings resulting in an increase in potency. Replacing the meta-chloro with an acetyl group provided a suitable solvent exposed exit vector that maintained affinity. A series of alkyl and PEG linkers of lengths varying from 6 to 17 atoms were then attached to a VHL ligand (Figure 6). The addition of a positively charged piperazinyl group lead to the identification of the SHP degrader SHP2-D26 which achieves DC_50_ values of 6 and 3 nM in esophageal cancer KYSE520 and acute myeloid leukemia MV4;11 cells respectively and can reduce SHP2 protein levels by >95% in cancer cells. The allosteric PROTAC SHP2-D26 was found to be 30 times more potent in inhibiting the phosphorylation of ERK and cell growth in KYSE520 and MV4;11 cancer cell lines than the allosteric inhibitor SHP099. Despite promising cellular activity these SHP2 PROTACs exhibit limited in vivo efficacy when dosed as a single agent (<20% tumor growth inhibition).^65^ Following this publication, Miao et al. have utilized an alternative exit vector to develop allosteric SHP2 PROTACs which show enhanced in vivo efficacy.^66^ By choosing to target SHP2 with an allosteric PROTAC these authors have built on the efficacy and physicochemical profile of the allosteric inhibitor, resulting in enhanced efficacy as afforded by the degradation phenotype.

**Figure 6:**
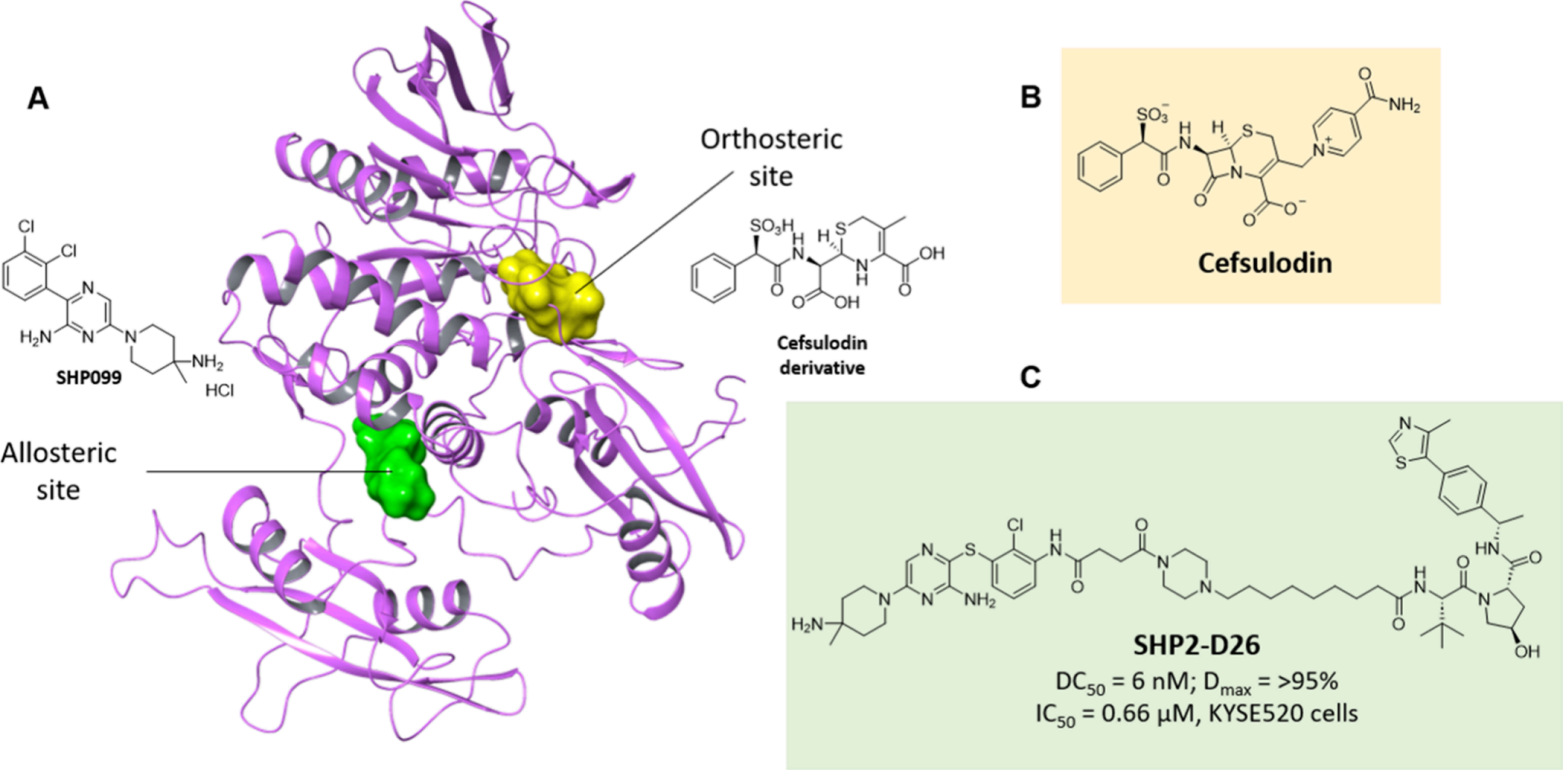
Allosteric SHP2 PROTACs. (A) Overlay of cocrystal structures SHP099 (green) in SHP2 (PDB 5EHR) with Cefsulodin derivative (yellow) which binds to the active site in SHP2 (PTP domain) (PDB 4RDD) (B) Chemical structure of Cefsulodin (C) Chemical structure of allosteric SHP2 inhibitor SHP099 and SHP2 PROTAC SHP2-D26.

### Physicochemical Property Improvement

PROTACs need to incorporate moieties that bind to both a target protein and to an E3 ligase. The resulting bifunctional molecules therefore usually sit well outside of the established “rule of 5” space used for decades by medicinal chemists to predict oral absorption. Despite existing outside this traditional space, many bifunctional degrader clinical candidates have shown that oral bioavailability is achievable for this compound class by careful optimization of their drug-like properties. As the field matures, biopharma companies at the forefront of these drug development efforts have begun to share their perspectives on what it takes to achieve orally bioavailable PROTACs. Through analysis of their PROTAC program databases, Arvinas^67^ and AstraZeneca^68^ have both published guidelines which suggest the physicochemical space a degrader should occupy to give the best chance of oral absorption. Interestingly, both companies reached similar conclusions that while there is some scope for expansion of acceptable ranges for other properties, the number of exposed/nonshielded hydrogen-bond donors should ideally be no more than two. For PROTAC drug developers, having a choice of target protein binding ligand can offer opportunities to tune degrader physicochemical profile. By considering the use of allosteric or nonfunctional ligands, drug hunters may be able to access ligands with more favorable properties for clinical development.

### TEAD

TEAD (transcriptional enhanced associate domain)­1–4 are transcription factors which are key components of the Hippo pathway. Binding of TEAD to its coactivators YAP (yes associated protein) or TAZ (transcriptional coactivator with PDZ binding motif) causes transcriptional activation. TEAD inhibitors are known, with chemical matter developed for both the TEAD/YAP or TAZ interface, and for an allosteric internal pocket, in which palmitoylation occurs.^69^ Additionally, reports describing the use of these inhibitors as target protein binders for bifunctional degraders have followed.^70,71^

Pobbati et al. were the first to demonstrate inhibition of TEAD proteins via ligand binding to the internal palmitoylation pocket.^72^ A computational druggability analysis predicted that the inner lipid pocket had the potential to bind small molecules with high affinity, by virtue of the pocket volume and electrostatic environment. By screening compound libraries consisting of fragments and FDA approved drugs, the authors identified Flufenemic acid (FA) as the top hit in a DSF screen. STD NMR and subsequent X-ray crystallography confirmed that Flufenemic acid bound to the palmitate pocket. Additionally, ligand electron density for FA was also observed at the shallow pocket at “interface 3” of the TEAD-YAP interface. Although in vitro characterization showed that FA does not abrogate YAP binding to TEAD, cellular assays indicated that binding of FA caused a decrease in TEAD transcriptional activity. Characterization of this internal pocket has had significant impact by inspiring the development of many TEAD lipid pocket inhibitors.^73^

TEAD-YAP/TAZ PPI disruptors have been disclosed by Novartis,^74^ which bind at “interface 3”, the interface which makes the largest contribution to overall binding between the TEAD-YAP proteins. Additionally, a patent detailing conjugation of these binders to a linker and CRBN E3-ligase binding moiety has been published.^75^ Researchers at Genentech have instead utilized a ligand developed for the TEAD allosteric palmitoylation pocket to generate proof of principle bifunctional degraders.^76^ Having been designed for different pockets on the TEAD proteins, these ligands have different physicochemical properties and therefore provide different chemical starting points for the development of TEAD PROTACs (Figure 7). As part of their guidelines, Arvinas have suggested a “budget” for prospective target protein binders. By considering the interface 3 and palmitoylation pocket binders in the context of this budget, it would suggest that PROTACs based on interface 3 binders would be more challenging to make orally bioavailable, due to their high molecular weight and an additional hydrogen bond donor.

**Figure 7:**
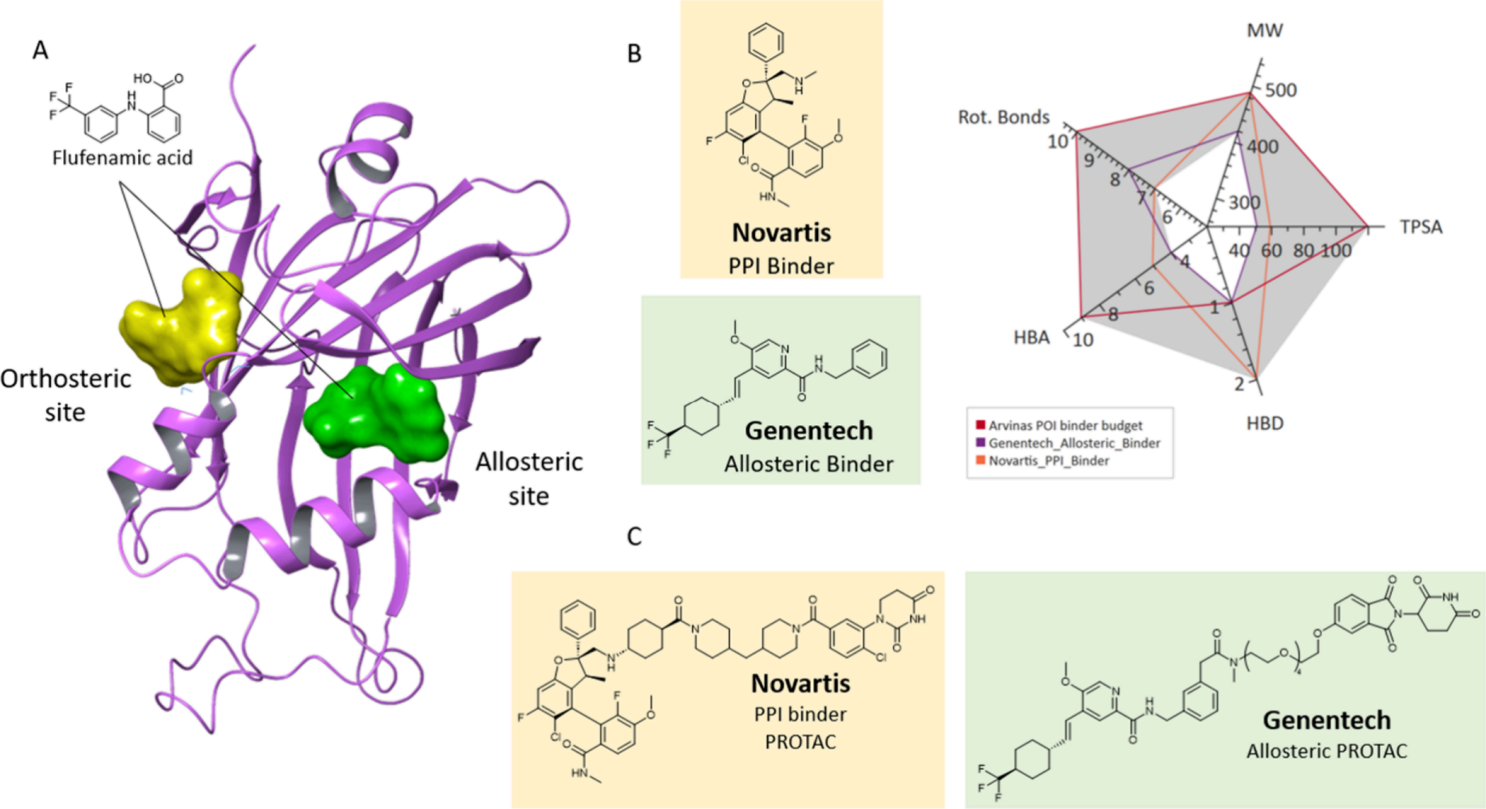
Identification of allosteric binding pocket within TEAD protein. (A) – Flufenamic acid binds to the allosteric pocket (green) and interface 3 pocket (yellow) of TEAD (5DQ8). B – Novartis and Genentech, utilize ligands that bind in the palmitoylation pocket and ‘interface 3′ respectively, with different physicochemical properties. C – Chemical structures of Novartis and Genentech bifunctional degraders made from their respective TEAD ligands.

Having a ligand with suitable potency and physicochemical properties is still a requirement for PROTAC development, and it remains difficult to achieve suitable drug-like properties for binders at orthosteric binding sites that are hydrophilic in nature. Likewise, compounds with multiple hydrogen bond donors are undesirable as target protein ligands when oral bioavailability of the resulting bifunctional molecule is required, considering the concomitant addition of at least one hydrogen bond donor in the E3 ligase binder.^67,68^ In these cases, allosteric or nonfunctional ligands can offer more balanced properties providing better starting point for development into PROTACs. Furthermore, comparison of orthosteric vs allosteric PROTACs for the same target can demonstrate the potential for compounds to occupy a different physicochemical property space, thus accelerating advancing candidates to the clinic.

### Allosteric PROTACs Can Target GPCRs

Allosteric PROTACs offer opportunities to target membrane-bound proteins for degradation by recruiting them via their intracellular domains. This includes GPCRs and other membrane receptors which normally are targeted from the exterior of the cell by small-molecules binding to their extracellular agonist/antagonist binding domain. Vercirnon is an oral antagonist for the GPCR CCR9 (C–C chemokine receptor type 9) that was investigated in phase 3 clinical trials for Crohn’s disease but did not show significant efficacy. Oswald et al. solved the X-ray crystal structure of Vercirnon with CCR9 to a 2.9Å resolution and discovered it binds to a conserved intracellular allosteric site on CCR9 in contrast to the much more commonly targeted extracellular orthosteric site (Figure 8).^77^ Binding at this site stabilizes the inactive conformation of the GPCR and sterically blocks binding of G-protein or β-arrestin. This conserved intracellular allosteric binding site has now been identified at several GPCRs. In the first proof of concept study to degrade a GPCR via the intracellular allosteric binding site, PROTACs with vercirnon attached to a VHL ligand via a triazole PEG linker were shown to modestly degrade CCR9 with excellent selectivity over the related chemokine receptors CCR2 or CCR7.^78^ It will be interesting to see further optimization of this compound and for the concept to be expanded to other GPCRs.

**Figure 8:**
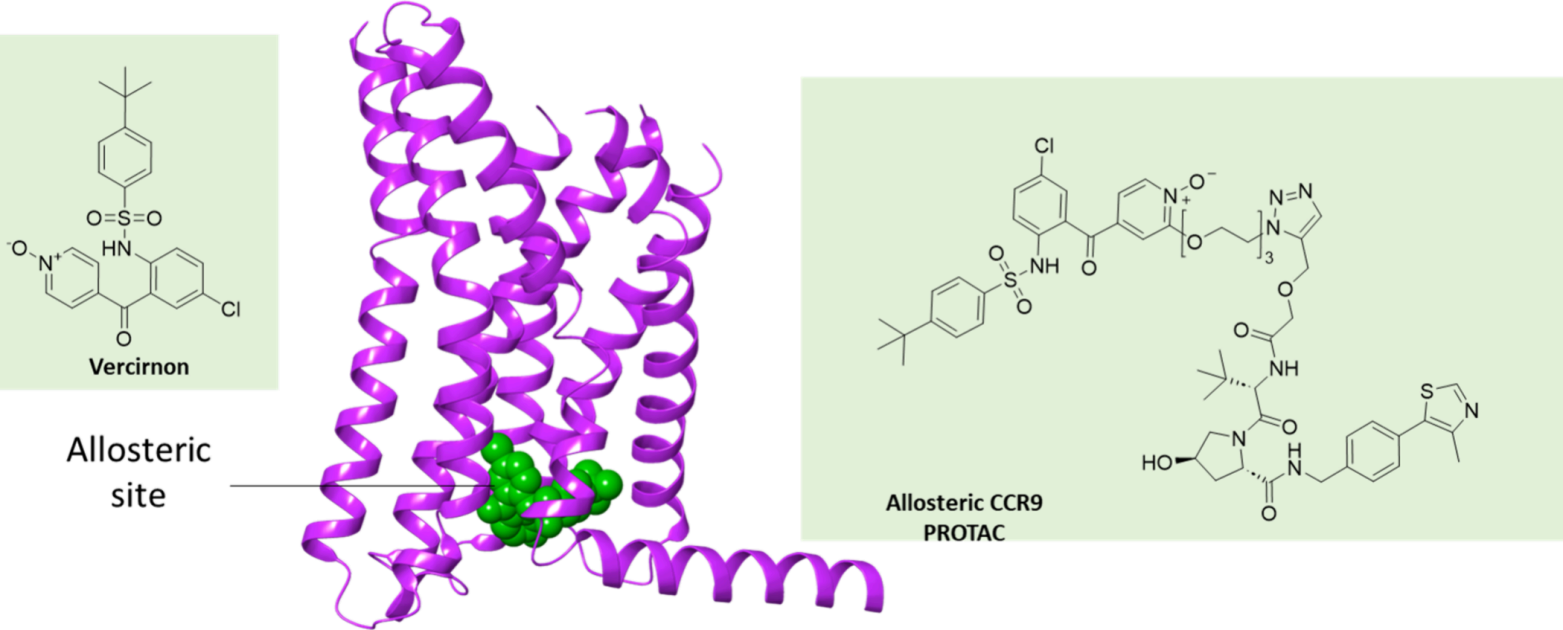
Vercirnon binds to an intracellular allosteric binding site on the GPCR CCR9, enabling the design of CCR9 PROTACs for targeted protein degradation.

### Using Nonfunctional Ligands to Expand Druggable Proteome

As well as targeting allosteric sites, PROTACs have been designed with ligands for sites that do not have a functional effect on their own. This is especially useful for difficult-to-drug proteins where binding ligands have been identified but were found to be nonfunctional as inhibitors.

### SMARCA2/4

The BAF chromatin remodelling complex is involved in many cellular processes relating to chromatin-dependent transcription and genes encoding many of its protein subunits are mutated in approximately 20% of human cancers. Farnaby et al. developed PROTACs to target the bromodomain of the ATPases SMARCA2 and SMARCA4, part of the larger BAF complex. SMARCA2/4 bromodomain inhibitors fail to phenocopy the antiproliferative effects observed by knockdown of SMARCA2 in cells lacking SMARCA4 activity and thus these bromodomain inhibitors are unable to be used to treat SMARCA4 mutant cancers. However, despite being nonfunctional binders, bromodomain inhibitors provide ideal starting points to the design of PROTACs. The collaborative team at the University of Dundee and Boehringer Ingelheim were able to design potent PROTAC degrader ACBI1 and demonstrate that rapid induced degradation of the SMARCA2 and SMARCA4 ATPases led to other BAF/PBAF subunits within these stable complexes being ejected from the BAF complex following SMARCA2/4 depletion.^79^ ACBI1 treatment was able to induce antiproliferative effects and apoptosis across multiple cancer cell lines, providing a case study for targeting a challenging multidomain protein, which had been difficult to drug via an inhibition approach. Although the site targeted may not have a functional effect in its own right via a small molecule inhibitor, the most ligandable domain can be selected for PROTAC development to achieve degradation of even large protein complexes.

Building on the discovery of ACBI1, optimization efforts were undertaken to improve target selectivity and oral bioavailability, to enable *in vivo* study. In this case it was necessary to redesign the target protein ligand, to give the resultant PROTAC a better chance of achieving oral exposure. In the development of PROTAC degrader ACBI2, the physicochemical properties of the target protein ligand were improved by reducing hydrogen bond donor count and increasing rigidity before pursuing degraders.^6^ The linker composition and exit vector placement was guided and rationalized by ternary cocrystal structures, yielding molecules which exhibit high potency and suitable physicochemical and pharmacokinetic properties to translate to oral *in vivo* efficacy, which has traditionally been difficult to achieve with VHL PROTACs (Figure 9). ACBI2 and its close analogue compound **7** demonstrated strong selectivity for the BAF (SWI/SNF) complex ATPase SMARCA2 over its highly similar paralogue SMARCA4 in human whole blood and showed consistent preferential degradation of SMARCA2 in all cell lines tested. Optimization of the physicochemical properties upfront of a nonfunctional ligand permitted the pharmacological evaluation of the synthetic lethality concept of selectively targeting SMARCA2 in SMARCA4-deficient cancers in vivo and in vitro.

**Figure 9:**
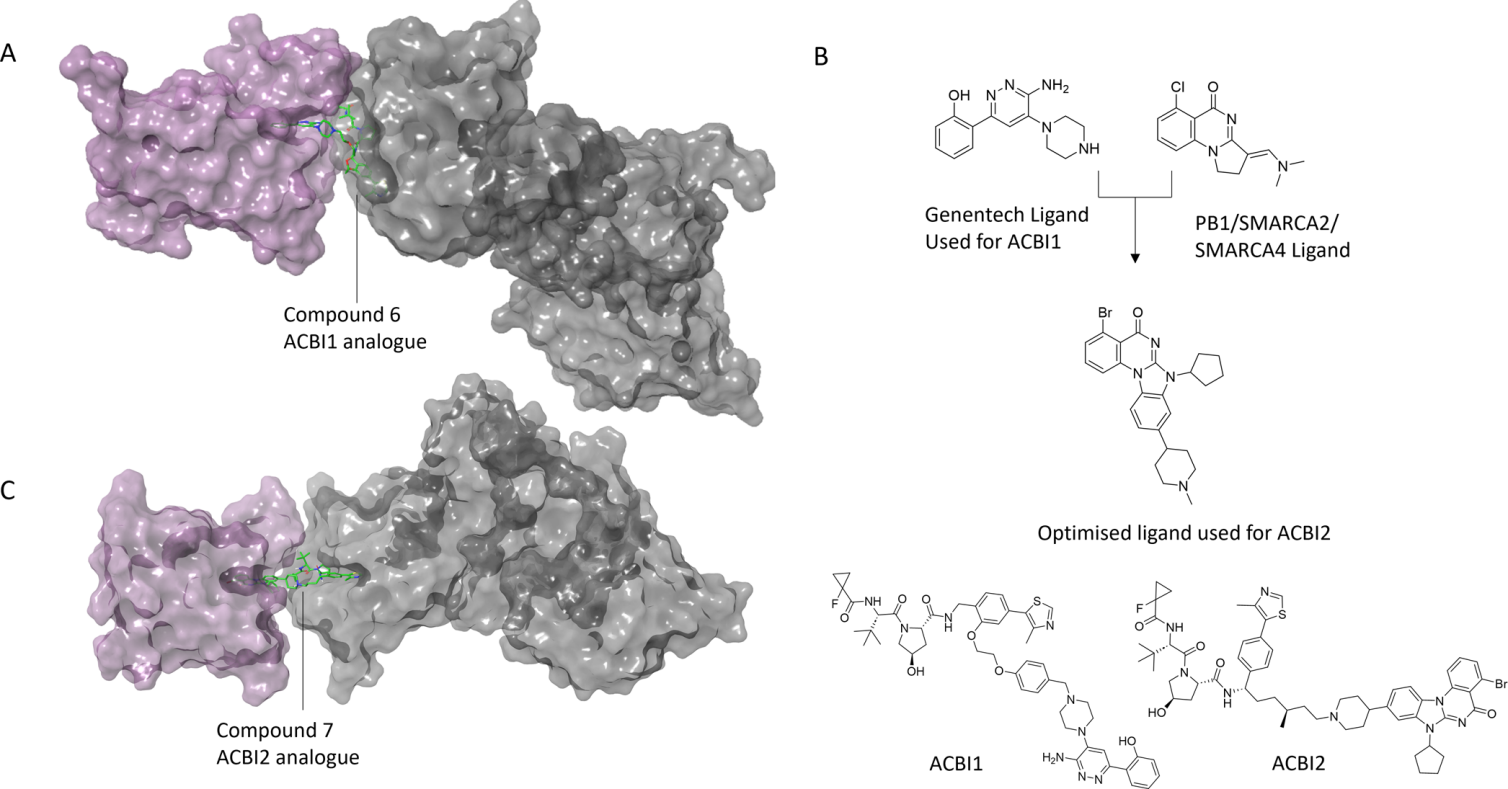
SMARCA2/4 PROTACs built on non-functional bromodomain ligands. (A) Ternary X-ray crystal structure of ACBI1 analogue bound to SMARCA2^BD^ (plum) and VHL (gray) (PDB 6HAY). (B) Ligand optimization for the development of ACBI2. (C) Ternary X-ray crystal structure of compound **7**, a close analogue of ACBI2, bound to SMARCA2^BD^ (plum) and VHL (gray) (PDB 7Z77).

More recently, smaller molecules built from the same SMARCA2/4 bromodomain ligands have been found and characterized to act as molecular glue degraders. These compounds covalently modify cysteine residues on the surface of the E3 Cullin-RING ligases (CRLs) CRL4^DCAF16^ and CRL1^FBX022^ substrate receptors from their solvent-exposed electrophilic pyridinyl-propargyl-amine “degradation tails”.^80,81^

### PCAF/GCN5

P300/CBP-associated factor (PCAF) and general control nonderepressible 5 (GCN5) are closely related epigenetic proteins, involved in many cellular processes. Humphrey’s et al. have reported the development of a chemical probe for the PCAF/GCN5 bromodomains which was optimized from a weakly potent, nonselective hit.^82^ They screened a set of ∼ 30,000 compounds containing known and potential acetyl-lysine mimetics in single shot format at 10 μM against PCAF using a fluorescence polarization assay with a labeled promiscuous bromodomain binder. Hits were progressed to full curve analysis, and orthogonal assays 15N–1H HSQC NMR and TR-FRET to confirm binding. This identified compound **8** which has low micromolar affinity and good ligand efficiency, which was developed through iterative medicinal chemistry cycles, guided by X-ray crystallography, into the potent and selective PCAF/GCN5 ligand GSK4027. However, chemical inhibition of the PCAF/GCN5 bromodomains was found to be insufficient to recapitulate the diminished inflammatory response of PCAF-knockout immune cells.

PROTACs remove all functions of the protein and are therefore able to better mimic the knockout phenotype. Bassi et al. have generated the first PROTACs able to degrade PCAF/GCN5, starting with the nonfunctional ligand GSK4027, which binds with similar affinity to PCAF and GCN5 but is selective over other bromodomain family members.^83^ Examination of the X-ray crystal structure of GSK4027 bound to human GCN5 bromo domain, shows the 4-position of the phenyl ring is solvent exposed, and this was used to attach a linker to thalidomide to make GSK983, a fast, potent, cell penetrant PCAF/GCN5 degrader (30 nM reduces both proteins by 80% in 10 min in PBMCs) (Figure 10). The individual isomers were synthesized, and the most active enantiomer was found to be (cis R,R) GSK699 which shows robust down-regulation of PCAF protein levels in macrophages and dendritic cells. Multiplex analysis of cytokine levels in the supernatants of LPS-stimulated cells revealed a marked reduction of IL-6, IL-12p70, IL-10, IL-1β, and IFN-γ production in both macrophages and DCs following treatment with GSK699 at 100 nM. This is a nice example of targeting difficult to drug multidomain proteins with a nonfunctional ligand and shows that the PROTAC approach can expand the druggable proteome to include nuclear epigenetic targets.

**Figure 10:**
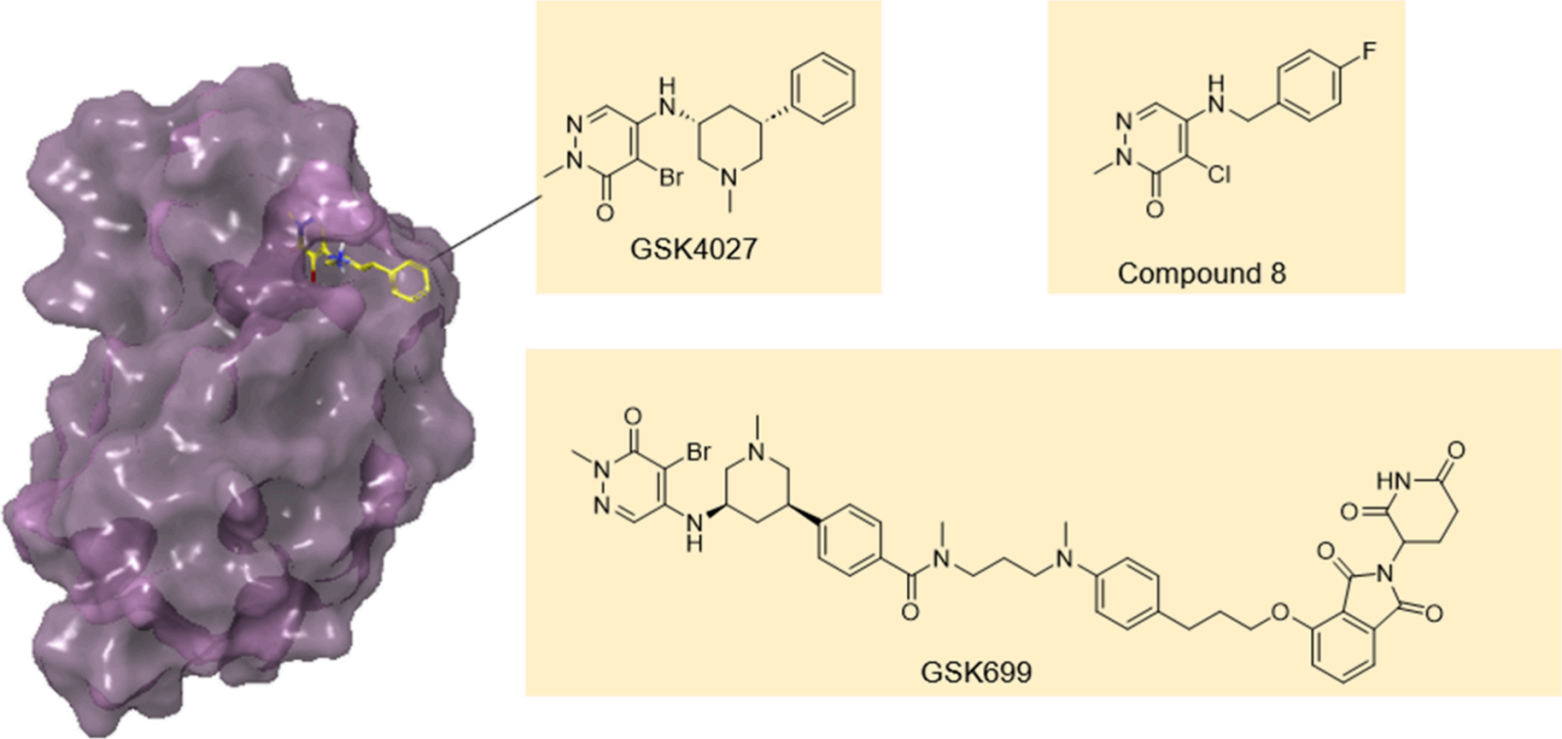
Developing PROTACs against PCAF/GCN5 from a nonfunctional ligand. The PCAF/GCN5 ligand compound **8**, identified from an acetyl lysine mimetic screen, was optimized to GSK4027. The X-ray crystal structure of GSK4027 bound to GCN5 (PDB 5MLJ) was used to identify the solvent exposed region to aid the design of PROTAC GSK699.

### FKBP51 (FK506-Binding Protein 51)

Tacrolimus is a powerful immunosuppressant discovered in 1984 by isolation from the culture broth of Streptomyces tsukubaensis, found in the soil near Tsukuba. Tacrolimus binds to FKBP12 and also FKBP51, which is a promising drug target for stress related mental disorders, chronic pain and obesity. Although tacrolimus binds to FKBP51, occupation of the binding site does not affect all of its functions. As binding site occupation does not lead to loss of function, Geiger et al. pursued degradation as a potential strategy to enable abrogation of FKBP51 scaffolding function.^84^ 220 PROTACs were synthesized using a combinatorial chemistry strategy from 15 different FKBP ligands, three E3 ligase ligands and five linker lengths, resulting in six FKBP51 degraders. Optimization, guided by biochemical characterization combined with binary and ternary X-ray crystallography resulted in the improved FKBP51 degrader SelDeg51, which spares FKBP12, due to negative cooperativity. Further investigation showed that SelDeg51 successfully abrogated the scaffolding function, whereas Tacrolimus occupation does not. The ternary X-ray structure of FKBP51:SelDeg51:VCB explains the observed positive cooperativity as the PROTAC appears to glue FKBP51 and the E3 ligase together (Figure 11). SelDeg51 efficiently depletes FKBP51 and reactivates glucocorticoid receptor-signaling, highlighting the enhanced efficacy of full protein degradation compared to classical FKBP51 binding.

**Figure 11:**
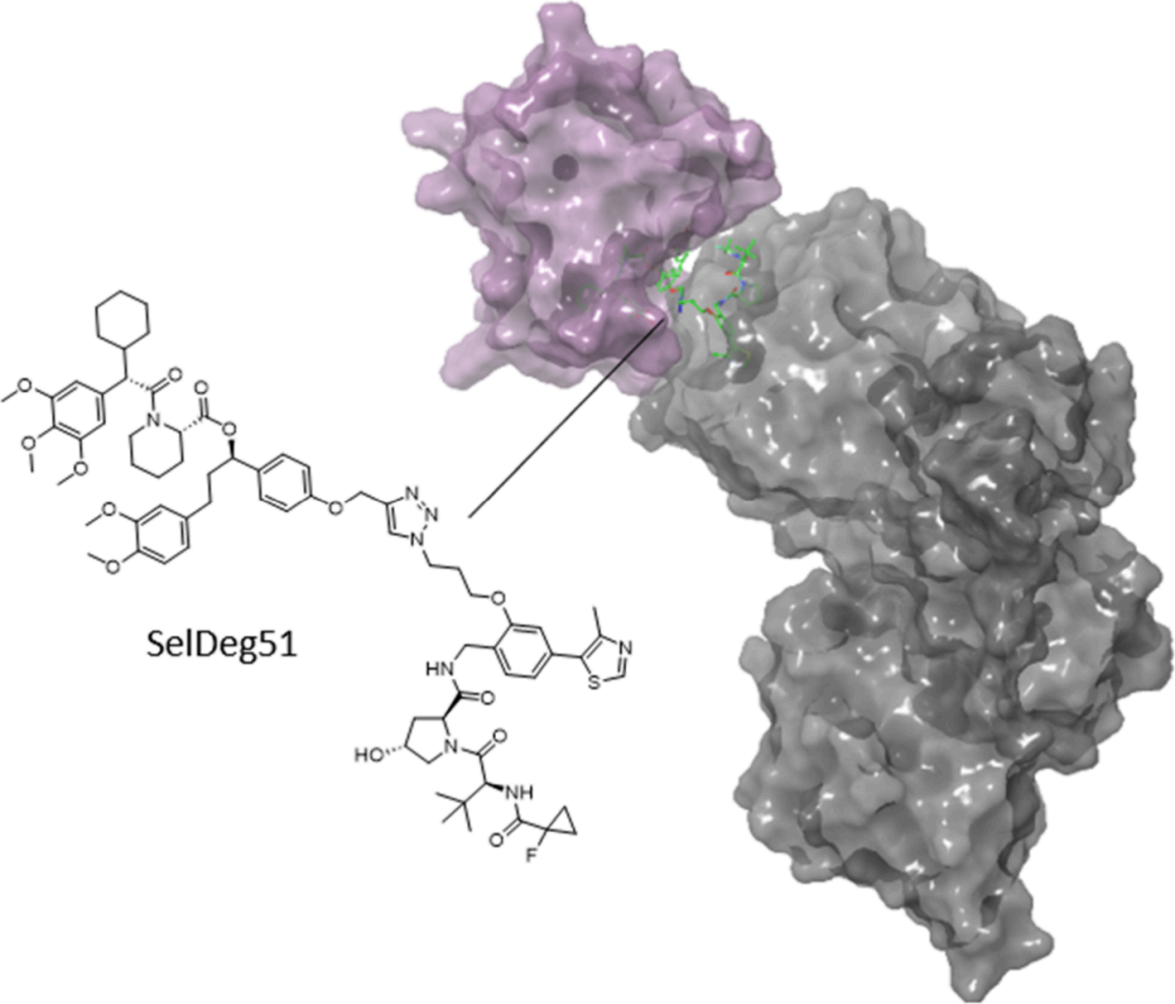
FKBP51 PROTACs synthesized from a nonfunctional ligand. Ternary X-ray crystal structure of FKBP51:SelDeg51:VCB (PDB 8PC2). The VHL and FKBP51 ligands are bound to their respective proteins with the linker solvent exposed, however, the 2-fluoro-2-cyclopropyl moiety on the VHL ligand is buried snugly between the two proteins and the FKP51 ligand interacts with VHL, resulting in positive cooperativity.

## Conclusions and Outlook

Bifunctional degraders have undoubtedly delivered many drug candidates to the benefit of patients, and the field could secure the first PROTAC FDA approval with Arvinas and Pfizer’s estrogen receptor (ER)-degrader vepdegestrant later this year. To date, however, PROTACs relying on orthosteric inhibitors as binders for the protein of interest remain over-represented in both the literature and the clinic. This bias reflects the historical prevalence of orthosteric ligands and their utility as tangible starting points for rational, modular PROTAC design. Yet, one of the most attractive features of the modality is its independence from functional sites—any sufficiently high-affinity binding surface can in principle serve as a degrader anchor. This raises the possibility of targeting proteins previously considered “undruggable.” In this Perspective, we have highlighted progress toward realizing this promise through PROTACs that exploit allosteric or nonfunctional ligands.

Despite the advantages of allosteric PROTACs, ligand availability remains a key bottleneck which makes their therapeutic development significantly more challenging than for orthosteric PROTACs. The design of degraders still follows a broadly “plug-and-play” strategy: a binder for the protein of interest is connected via a chemical linker to a binder for an E3 ligase. CRBN and VHL remain the most widely used ligases^85^ although other options, including IAP and DCAF1, have also been explored.^86,87^ While covalency has proven valuable in traditional inhibitor design, it is generally less advantageous for degraders when used at the target binding site, since irreversible engagement prevents rapid recycling and compromises the catalytic, substoichiometric nature of the modality.^88^

We envision that, now the initial PROTAC proof-of-concept has been clinically validated with orthosteric ligands for the target protein, drug discovery efforts will increasingly expand into the allosteric and functionally silent ligand space. Such ligands offer clear advantages: overcoming resistance at catalytic sites, improving physicochemical properties for drug development, and enabling intervention against protein targets whose active sites are intrinsically unsuitable for small-molecule binding. Importantly, this includes proteins rendered challenging by their structural features, such as protein–protein interaction interfaces and intrinsically disordered proteins. For example, AKT undergoes a conformational shift in its unphosphorylated form that restricts access to orthosteric inhibitors in KRAS or BRAF mutant settings, illustrating the need for nonorthosteric ligands to achieve efficacy across indications. Similarly, for kinases such as CRAF, structural studies suggest that chaperone and adaptor complexes (HSP90–CDC37, 14–3–3) maintain an autoinhibited conformation in cells, potentially rendering the kinase domain inaccessible, preventing engagement.^89,90,70^ Here, the absence of effective orthosteric degraders underscores the importance of pursuing alternative binding sites to unlock therapeutic potential in KRAS-driven cancers.^7,91,92^

Overcoming the hurdles associated with allosteric ligand development will also enable the realization of translational opportunities which extend well beyond degradation. Nonorthosteric ligands will be essential for the broader class of induced-proximity pharmacologies that recruit effectors to modulate protein function, including protein–protein interaction modulators to enhance target blockade (e.g., RevMed cyclophilin glues, RIPTACs) and small molecules that redirect enzymes to alter post-translational modifications or for gain-of-function pharmacology. Proof-of-principle studies using fusion proteins have demonstrated the ability to redirect acetylation, deacetylation, phosphorylation, dephosphorylation, and methylation of diverse substrates, including H3.3, RelA, p53, PDCD4, FOXO3a, Tau, AKT, ASK1, BRD4, and others.^93–98^ Realizing such concepts with small-molecule ligands will require access to suitable nonfunctional binders.

Bespoke development of allosteric and/or nonfunctional ligands will be required to capitalize on the promised potential of bifunctional allosteric proximity inducers. This presents unique challenges compared to the analogous orthosteric compounds, for which existing ligands are already available, and routes of ligand discovery well established. Identification of nonfunctional ligands is challenging as existing compound screening efforts are largely set up to detect compounds which elicit a biological response. Characterization of nonorthosteric binding sites via classical structural biology methods may run into issues in cases where construct design and purification is especially challenging, for example for complex multidomain proteins, PPI interfaces, or intrinsically disordered proteins.

A major challenge ahead, therefore, is to rethink how we discover ligands. Traditional paradigms have focused on functional activity as the primary readout, biasing discovery pipelines toward inhibitors or molecules active *per se*. For bifunctional molecules, however, ligands need only bind, not inhibit. Chemoproteomic approaches in live cells^99–101^ provide powerful means to generate interaction data sets in native contexts. DNA-encoded libraries (DELs) enable pooled screening of up to 10^12^ compounds, where the use of covalently attached DNA barcodes provides convenient starting point for linker attachment.^102^ Crystallographic fragment screens (e.g., XChem) can uncover binding sites across the entire protein surface.^103^ Advances in computational methods further promise to accelerate ligand discovery: virtual screening of vast chemical libraries^104^ machine learning-guided cofolding approaches such as AlphaFold-3 and Boltz,^105^ and AI-driven mapping of ligandable sites on structured and intrinsically disordered proteins.^99,106^ These and other machine-learning algorithms are expected to greatly benefit from global efforts to generate structure and binding data on protein–ligand complexes at scale, and protein–ligand interactions data sets.^107,108^ Parallel progress in modeling ternary complexes—integrating protein–protein docking with ab initio molecular dynamics—has enabled increasingly accurate predictions when binary complex liganded structures are available.^109–111^ Together, these experimental and computational innovations will help establish “binding-first” pipelines to deliver the nonorthosteric ligands required for next-generation proximity-inducing drugs.

The state of the art for allosteric PROTACs is still in its infancy but already demonstrates advantages over orthosteric approaches, including improved selectivity, efficacy, resistance management, and alternative physicochemical starting points. The future will see bespoke ligand discovery converge with advances in degradation and proximity-inducing modalities, extending beyond the ubiquitin–proteasome system into diverse cellular pathways. We are confident that the strategic development of nonorthosteric ligands will not only expand the degrader toolbox but also open entirely new frontiers in induced-proximity pharmacology. We expect that this will bring benefit to cancer patients with the potential to address resistance to current or emerging treatments, and other therapeutic areas beyond oncology.

## References

[1] Békés M., Langley D. R., Crews C. M. (2022). PROTAC targeted protein degraders:
the past is prologue. Nat. Rev. Drug Discovery.

[2] Vetma V., O’connor S., Ciulli A. (2025). Development of PROTAC Degrader Drugs
for Cancer. Annu. Rev. Cancer Biol..

[3] Teng M., Gray N. S. (2023). The rise of degrader
drugs. Cell
Chem. Biol..

[4] Burke M. R., Smith A. R., Zheng G. (2022). Overcoming Cancer Drug Resistance
Utilizing PROTAC Technology. Front Cell Dev
Biol..

[5] Zengerle M., Chan K. H., Ciulli A. (2015). Selective
Small Molecule Induced
Degradation of the BET Bromodomain Protein BRD4. ACS Chem. Biol..

[6] Kofink C. (2022). A selective and orally bioavailable VHL-recruiting
PROTAC achieves
SMARCA2 degradation in vivo. Nat Commun.

[7] Vetma V. (2024). Confounding Factors in Targeted Degradation of Short-Lived
Proteins. ACS Chem. Biol..

[8] Ou X., Gao G., Habaz I. A., Wang Y. (2024). Mechanisms of resistance to tyrosine
kinase inhibitor-targeted therapy and overcoming strategies. MedComm.

[9] Finlay M. R. V. (2014). Discovery of a potent
and selective EGFR inhibitor
(AZD9291) of both sensitizing and T790M resistance mutations that
spares the wild type form of the receptor. J.
Med. Chem..

[10] Wang S., Tsui S. T., Liu C., Song Y., Liu D. (2016). EGFR C797S
mutation mediates resistance to third-generation inhibitors in T790M-positive
non-small cell lung cancer. J Hematol Oncol.

[11] Jia Y. (2016). Overcoming EGFR­(T790M) and EGFR­(C797S) resistance with
mutant-selective
allosteric inhibitors. Nature..

[12] Jang J. (2020). Mutant-Selective Allosteric
EGFR Degraders are Effective Against
a Broad Range of Drug-Resistant Mutations. Angew.
Chem., Int. Ed. Engl..

[13] Niggenaber J. (2020). Complex Crystal Structures of EGFR with Third-Generation Kinase Inhibitors
and Simultaneously Bound Allosteric Ligands. ACS Med. Chem. Lett..

[14] Dillon M. (2021). Progress on Ras/MAPK
Signalling Research and Targeting in Blood and
Solid Cancers. Cancers (Basel)..

[15] Singhal A., Li B. T., O’Reilly E. M. (2024). Targeting
KRAS in cancer. Nat. Med..

[16] Neuzillet C. (2014). MEK in cancer and cancer
therapy. Pharmacol
Ther..

[17] Dudley D. T., Pang L., Decker S. J., Bridgest A. J., Saltiel A. R. (1995). A Synthetic
Inhibitor of the Mitogen-Activated Protein Kinase Cascade. Proc. Natl. Acad. Sci. U.S.A..

[18] Alessi D. R., Cuenda A., Cohen P., Dudley D. T., Saltiel A. R. (1995). PD 098059
is a specific inhibitor of the activation of mitogen-activated protein
kinase kinase in vitro and in vivo. J. Biol.
Chem..

[19] Bronte E. (2018). Cardiotoxicity mechanisms of the combination of BRAF-inhibitors and
MEK-inhibitors. Pharmacol Ther..

[20] Duncan K. E., Chang L. Y., Patronas M. (2015). MEK inhibitors:
A new class of chemotherapeutic
agents with ocular toxicity. Eye (Basingstoke)..

[21] Hu J. (2020). Potent and Selective
Mitogen-Activated Protein Kinase Kinase 1/2
(MEK1/2) Heterobifunctional Small-molecule Degraders. J. Med. Chem..

[22] Wei J. (2019). Discovery of a First-in-Class Mitogen-Activated Protein
Kinase Kinase
1/2 Degrader. J. Med. Chem..

[23] Vollmer S. (2020). Design, Synthesis, and
Biological Evaluation of MEK PROTACs. J. Med.
Chem..

[24] Iverson C. (2009). RDEA119/BAY 869766: A potent, selective, allosteric inhibitor of
MEK1/2 for the treatment of cancer. Cancer Res..

[25] Konopka J. B., Watanabe S. M., Witte O. N. (1984). An Alteration
of the Human C-Abl
Protein in K562 Leukemia Cells Unmasks Associated Tyrosine Kinase
Activity. Cell..

[26] Wong S., Witte O. N. (2004). The BCR-ABL story:
Bench to bedside and back. Annu. Rev. Immunol..

[27] Liu J. (2021). Recent advances in Bcr-Abl
tyrosine kinase inhibitors for overriding
T315I mutation. Chem. Biol. Drug Des..

[28] Cortes J. E. (2018). Ponatinib efficacy and
safety in Philadelphia chromosome-positive
leukemia: final 5-year results of the phase 2 PACE trial. Blood..

[29] Schoepfer J. (2018). Discovery of Asciminib (ABL001), an Allosteric
Inhibitor of the Tyrosine
Kinase Activity of BCR-ABL1. J. Med. Chem..

[30] Deeks E. (2022). D. Asciminib:
First Approval. Drugs..

[31] Wylie A. A. (2017). The allosteric inhibitor
ABL001 enables dual targeting of BCR-ABL1. Nature..

[32] Szankasi P., Schumacher J. A., Kelley T. W. (2016). Detection of BCR-ABL1 mutations that
confer tyrosine kinase inhibitor resistance using massively parallel,
next generation sequencing. Ann. Hematol..

[33] Eide C. A. (2019). Combining the Allosteric
Inhibitor Asciminib with Ponatinib Suppresses
Emergence of and Restores Efficacy against Highly Resistant BCR-ABL1Mutants. Cancer Cell.

[34] Deng X. (2010). Expanding the diversity
of allosteric Bcr-Abl inhibitors. J. Med. Chem..

[35] Yang Y. (2020). Global PROTAC Toolbox
for Degrading BCR-ABL Overcomes Drug-Resistant
Mutants and Adverse Effects. J. Med. Chem..

[36] Burslem G. M., Bondeson D. P., Crews C. M. (2020). Scaffold
hopping enables direct access
to more potent PROTACs with: In vivo activity. Chem. Commun..

[37] Burslem G. M. (2019). Targeting BCR-ABL1 in chronic myeloid leukemia by PROTAC-mediated
targeted protein degradation. Cancer Res..

[38] Lai A. C. (2016). Modular PROTAC Design
for the Degradation of Oncogenic BCR-ABL. Angew.
Chem., Int. Ed. Engl..

[39] Liu H. (2022). Discovery and characterization of novel potent BCR-ABL degraders
by conjugating allosteric inhibitor. Eur. J.
Med. Chem..

[40] O’Shea J. J. (2015). The JAK-STAT pathway: Impact on human disease and therapeutic intervention. Annu. Rev. Med..

[41] Schwartz D. M. (2017). JAK inhibition as a
therapeutic strategy for immune and inflammatory
diseases. Nat. Rev. Drug Discovery.

[42] Moslin R. (2017). Identification of imidazo­[1,2-*b*] pyridazine TYK2
pseudokinase ligands as potent and selective allosteric inhibitors
of TYK2 signalling. Medchemcomm..

[43] Kato J. Y., Korenaga S., Iwakura M. (2023). Discovery
of a potent and subtype-selective
TYK2 degrader based on an allosteric TYK2 inhibitor. Bioorg. Med. Chem. Lett..

[44] Lupardus P. J. (2014). Structure of the pseudokinase-kinase
domains from protein kinase
TYK2 reveals a mechanism for Janus kinase (JAK) autoinhibition. Proc. Natl. Acad. Sci. U. S. A..

[45] Landel I., Quambusch L., Depta L., Rauh D. (2020). Spotlight on AKT: Current
Therapeutic Challenges. ACS Med. Chem. Lett..

[46] Erickson E. C. (2024). Multiomic profiling
of breast cancer cells uncovers stress MAPK-associated
sensitivity to AKT degradation. Sci. Signal..

[47] You I. (2020). Discovery of an AKT
Degrader with Prolonged Inhibition of Downstream
Signaling. Cell Chem. Biol..

[48] Yu X. (2021). Design, Synthesis, and
Evaluation of Potent, Selective, and Bioavailable
AKT Kinase Degraders. J. Med. Chem..

[49] Yu X. (2022). Discovery of Potent,
Selective, and in Vivo Efficacious AKT Kinase
Protein Degraders via Structure-Activity Relationship Studies. J. Med. Chem..

[50] Zhu C. L. (2022). Structure-based rational design enables efficient discovery
of a
new selective and potent AKT PROTAC degrader. Eur. J. Med. Chem..

[51] Xu J. (2021). AKT Degradation Selectively Inhibits the Growth of
PI3K/PTEN Pathway-Mutant
Cancers with Wild-Type KRAS and BRAF by Destabilizing Aurora Kinase
B. Cancer Discovery.

[52] Yu X. (2022). Novel Allosteric Inhibitor-Derived
AKT Proteolysis Targeting Chimeras
(PROTACs) Enable Potent and Selective AKT Degradation in KRAS/BRAF
Mutant Cells. J. Med. Chem..

[53] Wu W. I. (2010). Crystal structure of
human AKT1 with an allosteric inhibitor reveals
a new mode of kinase inhibition. PLoS One..

[54] Shaw A. L. (2023). ATP-competitive and
allosteric inhibitors induce differential conformational
changes at the autoinhibitory interface of Akt1. Structure.

[55] Davies T. G. (2007). A Structural Comparison
of Inhibitor Binding to PKB, PKA and PKA-PKB
Chimera. J. Mol. Biol..

[56] Ashwell M. A. (2012). Discovery and optimization
of a series of 3-(3-phenyl-3H-imidazo­[4,5-b]
pyridin-2-yl)­pyridin-2-amines: Orally bioavailable, selective, and
potent ATP-independent Akt inhibitors. J. Med.
Chem..

[57] Yu X. (2022). Novel Allosteric Inhibitor-Derived
AKT Proteolysis Targeting Chimeras
(PROTACs) Enable Potent and Selective AKT Degradation in KRAS/BRAF
Mutant Cells. J. Med. Chem..

[58] Matozaki T., Murata Y., Saito Y., Okazawa H., Ohnishi H. (2009). Protein tyrosine
phosphatase SHP-2: A proto-oncogene product that promotes Ras activation. Cancer Science..

[59] Mainardi S. (2018). SHP2 is required for
growth of KRAS-mutant non-small-cell lung cancer
in vivo letter. Nat. Med..

[60] Ruess D. A. (2018). Mutant KRAS-driven cancers
depend on PTPN11/SHP2 phosphatase. Nat. Med..

[61] Zeng L. F. (2014). Therapeutic potential
of targeting the oncogenic SHP2 phosphatase. J. Med. Chem..

[62] Garcia
Fortanet J. (2016). Allosteric Inhibition of SHP2: Identification of a
Potent, Selective, and Orally Efficacious Phosphatase Inhibitor. J. Med. Chem..

[63] Chen Y. N. P. (2016). Allosteric inhibition of SHP2 phosphatase inhibits
cancers driven by receptor tyrosine kinases. Nature..

[64] Wang M., Lu J., Wang M., Yang C. Y., Wang S. (2020). Discovery of SHP2-D26
as a First, Potent, and Effective PROTAC Degrader of SHP2 Protein. J. Med. Chem..

[65] Deng Y. (2022). Therapeutic efficacy of the novel SHP2 degrader SHP2-D26,
alone or
in combination, against lung cancer is associated with modulation
of p70S6K/S6, Bim and Mcl-1. Cancer Gene Ther..

[66] Miao J. (2023). Discovery of a SHP2
Degrader with In Vivo Anti-Tumor Activity. Molecules..

[67] Hornberger K. R., Araujo E. M. V. (2023). Physicochemical
Property Determinants of Oral Absorption
for PROTAC Protein Degraders. J. Med. Chem..

[68] Schade M. (2024). Structural and Physicochemical
Features of Oral PROTACs. J. Med. Chem..

[69] Pobbati A. V., Kumar R., Rubin B. P., Hong W. (2023). Therapeutic targeting
of TEAD transcription factors in cancer. Trends
Biochem. Sci..

[70] Li H. (2024). Design,
Synthesis, and Bioevaluation of Transcriptional Enhanced
Associated Domain (TEAD) PROTAC Degraders. ACS
Med. Chem. Lett..

[71] Lu Y. (2025). Selective Degradation
of TEADs by a PROTAC Molecule Exhibited Robust
Anticancer Efficacy In Vitro and In Vivo. J.
Med. Chem..

[72] Pobbati A. V. (2015). Targeting the Central Pocket in Human Transcription Factor TEAD as
a Potential Cancer Therapeutic Strategy. Structure..

[73] Lou J. (2022). A chemical perspective
on the modulation of TEAD transcriptional
activities: Recent progress, challenges, and opportunities. Eur. J. Med. Chem..

[74] Furet P. (2022). The First Class of Small Molecules Potently Disrupting
the YAP-TEAD
Interaction by Direct Competition. ChemMedChem.

[75] Chapeau, E. Bifunctional degraders comprising a TEAD binder WO2023031801A1 2023

[76] Pham T. H. (2024). Targeting the Hippo
pathway in cancers via ubiquitination dependent
TEAD degradation. eLife..

[77] Oswald C. (2016). Intracellular allosteric antagonism of the CCR9 receptor. Nature..

[78] Huber M. E. (2022). A Chemical Biology Toolbox Targeting the Intracellular Binding Site
of CCR9: Fluorescent Ligands, New Drug Leads and PROTACs. Angew. Chem., Int. Ed..

[79] Farnaby W. (2019). BAF complex vulnerabilities in cancer demonstrated via structure-based
PROTAC design. Nat. Chem. Biol..

[80] Spiteri, V. A. Dual E3 ligase recruitment by monovalent degraders enables redundant and tuneable degradation of SMARCA2/4 bioRxiv. 2025 10.1101/2025.08.04.668513.

[81] Villemure E. (2025). Rational design of potent
small molecule SMARCA2/A4 (BRM/BRG1) degraders
acting via the recruitment of FBXO22. Nat Commun.

[82] Humphreys P. G. (2017). Discovery of a potent, cell penetrant, and
selective p300/CBP-associated
factor (PCAF)/general control nonderepressible 5 (GCN5) bromodomain
chemical probe. J. Med. Chem..

[83] Bassi Z. I. (2018). Modulating PCAF/GCN5
Immune Cell Function through a PROTAC Approach. ACS Chem. Biol..

[84] Geiger T. M. (2024). Discovery of a Potent Proteolysis Targeting
Chimera Enables Targeting
the Scaffolding Functions of FK506-Binding Protein 51 (FKBP51). Angew. Chem., Int. Ed..

[85] Ishida T., Ciulli A. (2021). E3 Ligase Ligands for PROTACs: How They Were Found
and How to Discover New Ones. SLAS Discovery.

[86] Schröder M. (2024). DCAF1-based PROTACs
with activity against clinically validated targets
overcoming intrinsic- and acquired-degrader resistance. Nat Commun.

[87] Mares A. (2020). Extended pharmacodynamic
responses observed upon PROTAC-mediated
degradation of RIPK2. Commun Biol.

[88] London N. (2025). Covalent Proximity
Inducers. Chem. Rev..

[89] García-Alonso S. (2022). Structure
of the RAF1-HSP90-CDC37 complex reveals the basis of RAF1
regulation. Mol. Cell.

[90] Molzan M., Ottmann C. (2012). Synergistic binding of the phosphorylated
S233- and
S259-binding sites of C-RAF to One 14–3-3ζ dimer. J. Mol. Biol..

[91] Sanclemente M. (2018). c-RAF Ablation Induces Regression of Advanced
Kras/Trp53 Mutant Lung
Adenocarcinomas by a Mechanism Independent of MAPK Signaling. Cancer Cell.

[92] Sanclemente M. (2021). RAF1 kinase activity
is dispensable for KRAS/p53 mutant lung tumor
progression. Cancer Cell..

[93] Wang W. W. (2021). Targeted Protein Acetylation
in Cells Using Heterobifunctional Molecules. J. Am. Chem. Soc..

[94] Chen P. H. (2021). Modulation of Phosphoprotein Activity by Phosphorylation Targeting
Chimeras (PhosTACs). ACS Chem. Biol..

[95] Hu Z. (2023). Targeted Dephosphorylation
of Tau by Phosphorylation Targeting Chimeras
(PhosTACs) as a Therapeutic Modality. J. Am.
Chem. Soc..

[96] Siriwardena S. U. (2020). Phosphorylation-Inducing Chimeric Small Molecules. J. Am. Chem. Soc..

[97] Zhang Q. (2023). Protein Phosphatase 5-Recruiting Chimeras for Accelerating Apoptosis-Signal-Regulated
Kinase 1 Dephosphorylation with Antiproliferative Activity. J. Am. Chem. Soc..

[98] Seabrook L. J. (2024). Methylarginine targeting chimeras for lysosomal degradation of intracellular
proteins. Nat. Chem. Biol..

[99] Offensperger F. (2024). Large-scale chemoproteomics
expedites ligand discovery and predicts
ligand behavior in cells. Science.

[100] Backus K. M. (2016). Proteome-wide covalent ligand discovery
in
native biological systems. Nature..

[101] Parker C. G. (2017). Ligand and Target Discovery
by Fragment-Based
Screening in Human Cells. Cell.

[102] Peterson A. A., Liu D. R. (2023). Small-molecule discovery
through
DNA-encoded libraries. Nat. Rev. Drug Discovery.

[103] Fearon D. (2025). Accelerating Drug Discovery
With High-Throughput Crystallographic
Fragment Screening and Structural Enablement. Appl. Res..

[104] Lyu J., Irwin J. J., Shoichet B. K. (2023). Modeling
the expansion of virtual
screening libraries. Nat. Chem. Biol..

[105] Nittinger E., Yoluk Ö., Tibo A., Olanders G., Tyrchan C. (2025). Co-folding, the future of docking
– prediction
of allosteric and orthosteric ligands. Artif
Intell Life Sci..

[106] Wu K. (2025). Design of intrinsically disordered region binding
proteins. Science.

[107] Edwards A. M. (2025). Protein–ligand data at scale to support
machine learning. Nat. Rev. Chem..

[108] Durairaj, J. PLINDER: The protein-ligand interactions dataset and evaluation resource. bioRxiv, 2025 10.1101/2024.07.17.603955.

[109] Jofily P., Kalyaanamoorthy S. (2025). P4ward: An Automated Modeling Platform
for Protac Ternary Complexes. J. Chem. Inf Model..

[110] Drummond M. L., Henry A., Li H., Williams C. I. (2020). Improved
accuracy for modeling ProTAC-mediated ternary complex formation and
targeted protein degradation via new in silico methodologies. J. Chem. Inf Model..

[111] Dixon T. (2022). Predicting the structural basis of targeted
protein
degradation by integrating molecular dynamics simulations with structural
mass spectrometry. Nat. Commun..

